# Percutaneous Embolization of No Ligated Vertical Veins After Total Anomalous Pulmonary Vein Return Operation and Risk Factors for Its Persistence

**DOI:** 10.3390/jcdd11120393

**Published:** 2024-12-07

**Authors:** Stasa Krasic, Sofija Popovic, Vesna Topic, Mila Stajevic, Ivan Dizdarevic, Sasa Popovic, Dejan Nesic, Vladislav Vukomanovic

**Affiliations:** 1 Cardiology Department, Mother and Child Health Institute of Serbia, 11070 Belgrade, Serbia; 2 Faculty of Medicine, University of Belgrade, 11129 Belgrade, Serbia; 3 Radiology Department, Mother and Child Health Institute of Serbia, 11070 Belgrade, Serbia; 4 Cardiac Surgery Department, Mother and Child Health Institute of Serbia, 11070 Belgrade, Serbia; 5 Institute of Medical Physiology, Faculty of Medicine, University of Belgrade, Visegradska 26/II, 11129 Belgrade, Serbia

**Keywords:** total anomalous pulmonary venous return, vertical vein, percutaneous embolization, left atrium diameter

## Abstract

Background: The vertical vein (VV) ligation during the total anomalous pulmonary venous return (TAPVR) correction is still controversial. Our study aimed to define the potential risk factors for VV persistence and their percutaneous occlusion. Methods: The retrospective cohort study included 40 patients (26 males) with TAPVR treated at the tertiary referral center from 2005 to 2024. Results: The average days of age at diagnosis was two (IQR 1–8). Complex congenital heart disease with TAPVR was diagnosed in eight patients. A supracardiac type of TAPVR was found in 47% of them. The patients underwent the operation on their eighth day of life (IQR 5–57). The follow-up period was 32 months (IQR 8–99). The early postoperative mortality rate was 17.5%, significantly frequent in the patients’ group with combined CHD (*p* = 0.002). Four were reoperated on—three due to a postoperative obstruction between the pulmonary venous confluence and the left atrium (LA), while in one patient, a redirection of the VCI was performed. Four patients, aged 12.3 on average (IQR 8.9–14.7), underwent vertical vein embolization. All patients achieved complete occlusion with AVP2. The LA diameter Z score was lower than −4, an increased risk for VV persistence of almost 19 times (OR 18.6, 95% CI 1.6–216.0). Conclusions: We found that an LA diameter Z score of lower than −4 was a major risk factor for VV persistence. Percutaneous VV embolization is a safe and effective procedure in adolescents.

## 1. Introduction

The total anomalous pulmonary venous return (TAPVR) is a congenital heart disease (CHD) accounting for up to 1–3% of all birth heart defects. According to the Darling classification, four TAPVR types exist: (1) supracardiac (45–55% of cases); (2) cardiac (20–30% of cases); (3) infracardiac or infradiaphragmatic (13–25% of cases); (4) mixed (5% of all cases). On the other hand, Smith’s classification considers the hemodynamic impact of the cardiac anomaly—non-obstructive and obstructive types [1,2]. A clinical presentation is considered by the obstruction degree; the more pronounced the obstruction, the more dramatic the clinical manifestation at an earlier age [3].

The prognosis can be abysmal without intervention, with a mortality rate of 80% during infancy [4]. The TAPVR treatment is surgical, depending on the TAPVR type and associated cardiac malformations. The surgical technique implies an anastomosis between the left atrium (LA) and pulmonary veinous confluence (PVC), either directly or by creating a new left atrium (the sutureless technique) and ASD closure [5]. Ligation of the vertical vein (VV) is still controversial, while hepatic ischemia has been described in VV ligation in patients with type III TAPVR [6]. On the other hand, a patent VV may temporarily redistrict pulmonary venous blood in noncompliant left heart chambers until they can grow and adapt to the increased flow demands. After the compliance improvement, the spontaneous close of VV due to preferential flow to the left atrium is expected [6,7].

Persistent VV is more common in type 1 TAPVR than type 3 due to the high resistance of the hepatic capillary bed [8]. A patent VV presents as a pretricuspid shunt leading to right heart dilatation [8]. The risk factors for the persistence of VV after a TAPVR operation have not yet been defined.

The primary aims of our study were to define the potential risk factors for VV persistence and recanalization if it was not ligated and to evaluate the technique of percutaneous VV occlusion. The secondary aims were to assess short- and long-term outcomes in our patients with TAPVR.

## 2. Materials and Methods

The retrospective cohort study included all the patients with TAPVR treated at the tertiary referral center for congenital heart disease and reflected a period of 19 years (from 2005 to 2024). In some patients, the diagnosis was established by echocardiography and confirmed by CT angiography.

An echocardiography examination was performed upon admission to establish a diagnosis of TAPVR. The following details were identified: the existence of a TAPVR, the TAPVR type, obstruction, and the associated cardiovascular lesions. Preoperative pulmonary venous obstruction was defined as a peak pulmonary venous pathway Doppler velocity of >2 m/s or atrial level obstruction to the left ventricle (LV) filling as a peak right-to-left atrial peak Doppler velocity of >1 m/s. The patients with obstructed TAPVR had VV obstruction with marked pulmonary hypertension, developed critical cyanosis and hypoxemia soon after birth and fell into cardiogenic shock. Subsequently, emergency surgical repair is the only way to save them.

Surgical correction was performed using a standard approach through a medial sternotomy. If the LA was poorly developed, the anastomosis was extended into the right atrium, and the patch was repositioned to the right between the PVC-LA anastomosis and the inflows of the caval veins (SVC and IVC). After releasing the clamp, the VV was ligated. The VV ligation had not been performed before 2017, but after 2017, the operative technique was changed, and the surgeons started to ligate it. The operative technique was altered, considering VV persistence was registered during the follow-up period.

Demographic parameters, clinical presentation, and echocardiography examination were evaluated in all children before the surgical correction to define risk factors for the persistence or recanalization of the VV. M-mode, 2-D echocardiography, and Detroit z-score estimations measured the LA and LV end-diastolic diameters [9].

**Figure 1:**
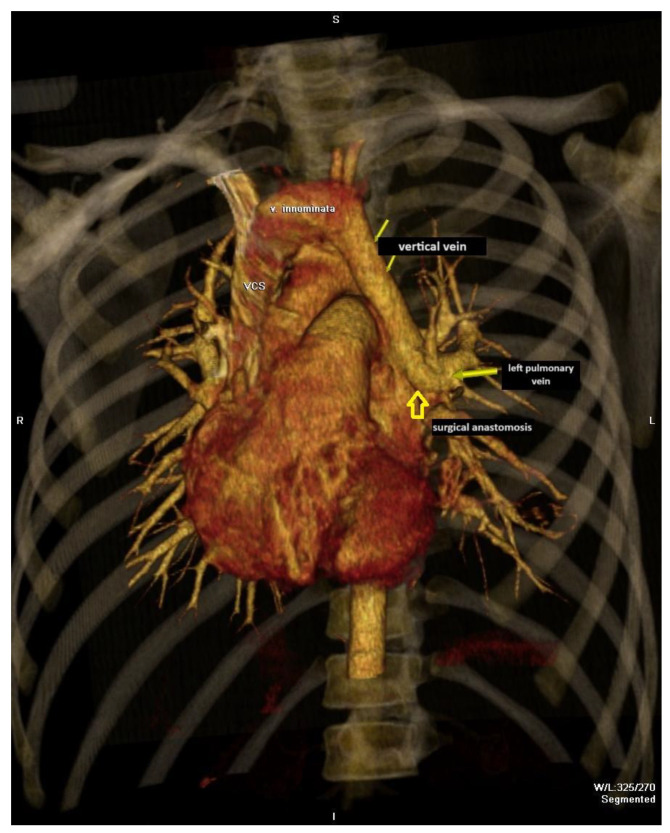
A 3D reconstruction of the CT angiography in the patient with persistent vertical vein after total anomalous pulmonary vein return surgery.

**Figure 2:**
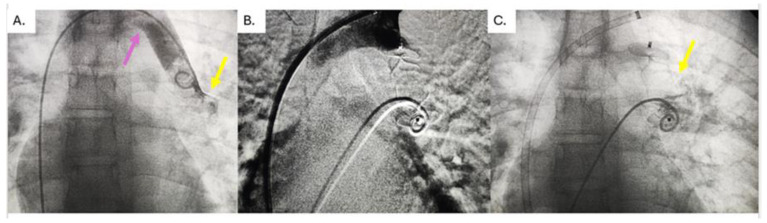
(**A**) An angiography of the vertical vein; the yellow arrow highlights the inflow of the left upper pulmonary vein, the pink arrow highlights the inflow of the vertical vein in the bridging vein; (**B**) an Amplatzer vascular plug 2 placed into the vertical vein, but still connected with the cable; (**C**) retrieved Amplatzer vascular plug 2 into the vertical vein.

During the follow-up period, serial echocardiography examinations were performed. In some patients, echocardiography showed VV persistence with right heart chamber volume overload (Figure 1). In those patients with persistent or recanalization VV, CT angiography with 3D reconstruction was performed (Figure 2). The horizontal (the narrowest and the largest) and vertical (from the pulmonary vein to the left innominate vein (LIV)) diameters of VV were estimated—for the landing zone.

### 2.1. Vertical Vein Occlusion

**Figure 3:**
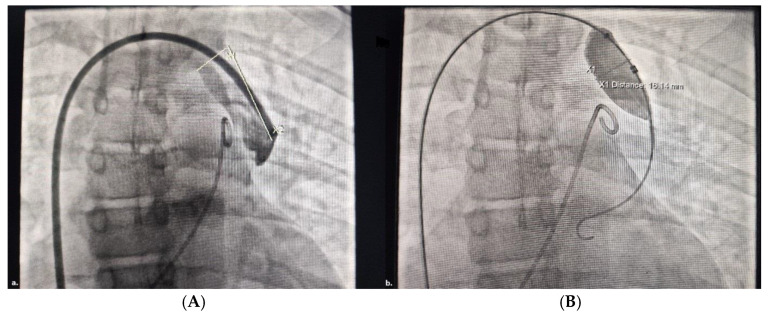
(**A**) Marcations of the landing zone; (**B**) balloon test occlusion—an Amplatzer sizing balloon was placed and inflated into the vertical vein, while a pigtail catheter was placed into the pulmonary artery.

Cardiac catheterization was performed under general endotracheal anesthesia. Both femoral veins and the left jugular vein were cannulated. A pigtail catheter was placed via the right femoral vein into the pulmonary artery. Then, a pigtail catheter was placed through the vertical vein into the left atrium (Figure 2A). Manometric and oximetric tests using the pull-back technique were performed to estimate the ratio between blood flow through systemic and pulmonary circulation (Qp:Qs). After the pulmonary artery (PA) and VV angiography, the right positions of the left pulmonary vein inflow in VV and the connection of VV with LIV were estimated (landing zone) (Figure 3A).

The balloon occlusion test was performed using an Amplatzer Sizing Balloon II (Figure 3B). The balloon was introduced through the right femoral vein in a 9-Fr short introducer, advanced on an exchange wire to the “landing zone”, and then inflated with diluted contrast media for total occlusion. Another pigtail catheter was introduced into the pulmonary artery through the left femoral vein. The wedge pressure in PA before and after 10 min of the balloon test occlusion was measured.

If the PA pressure was not elevated after the balloon test occlusion, we obtained access to VV occlusion. We used an Amplatzer Vascular Plug 2 (AVP2). The devices were chosen based on the landing zone diameters. The device size was 40% larger than the narrowest VV diameter. Before releasing the device, a control angiography was performed over the left jugular vein and pulmonary artery to confirm the device’s position (Figure 2B). In contrast, obstruction of the systemic veins was ruled out, and PV infusion into the LA was confirmed (Figure 2C). Ten minutes after the device’s release, a control angiography in the PA in the venous phase confirmed the absence of flow through the vertical vein and adequate perfusion of the pulmonary veins. After the intervention, acetyl salicylate acid was started in an antiaggregation dose.

### 2.2. Statistics

Basic (descriptive) statistics included mean values, standard deviations, and the median and interquartile range of monitored parameters. Furthermore, the difference in the distribution of certain features among the tested groups was determined using the χ2 or Fisher’s test. The normality of the distribution of the numerical variables was tested using the Shapiro–Wilk and Kolmogorov–Smirnov tests. The groups were compared using the Student’s *t*-test and Mann–Whitney test. Binomial logistic regression analysis was used to explain the relationship between dependent binary variables and independent variables. All the statistical methods were significant if the *p*-value was ≤0.05. Data processing was carried out using the statistical software SPSS 25.0 for Windows 10.

## 3. Results

The retrospective cohort study included 40 patients (26 males), with an average days of age at the time of diagnosis of 2 (IQR 1–8; min–max: 1–570 days). Complex congenital heart disease with TAPVR was diagnosed in eight patients (20%)—5 had atrioventricular (AV) septal defect or inlet ventricular septal defect (VSD) with hypoplastic left ventricle (1/5 patient); one had a univentricular heart with great arteries transposition (Table 1). A supracardiac type of TAPVR was found in almost half of the patients (19/40 pts; 47.5%), while the mixed type was detected in 4/40 patients. Vertical vein obstruction was described in 25% of patients, more commonly in males (*p* = 0.07) and in patients with infracardiac TAPVR type (*p* = 0.03).

Echocardiography examination at the admission relieved low LV EDD (Z score −3.8, IQR −4.97–−1.32) and LA diameter (Z score −3.1, IQR −4.7–−2.2) Z scores. The ASD was measured at a median of 4 mm (IQR 2–6.5 mm). Tricuspid insufficiency was mild (+1, IQR 0.5–1), with right ventricular pressure (peak pressure gradient (PG)) of 38 mmHg, IQR 32–56 (Table 2).

The patients underwent the operation on their eighth day of life (IQR 5–57; min–max: 2–600), with a median body weight of 3.3 kg (IQR 2.8–4.0; min–max: 2–10). The patients with obstructive TAPVR were younger during the operation than the rest of the cohort (3, IQR 2–5 vs. 13, IQR 7–91 days of life; *p* < 0.001). A side-to-side anastomosis was performed on 25 patients, coronary sinus redirection on three, and the Warder procedure on one. Vertical vein ligation was performed on 13 patients. For patients with complex congenital heart disease, modified Blalock Taussig shunts (MBTS) were created in three patients, Glenn anastomosis in one and Damus-Keye-Stensel in one. The early postoperative mortality rate was 17.5% (7/40), significantly higher in the patients’ group with combined CHD (5/7; *p* = 0.002). The mortality and reoperation rates were not higher in patients with obstructive TAPVR than the rest of the cohort (*p* = 1.0 and *p* = 0.65, respectively).

**Figure 4:**
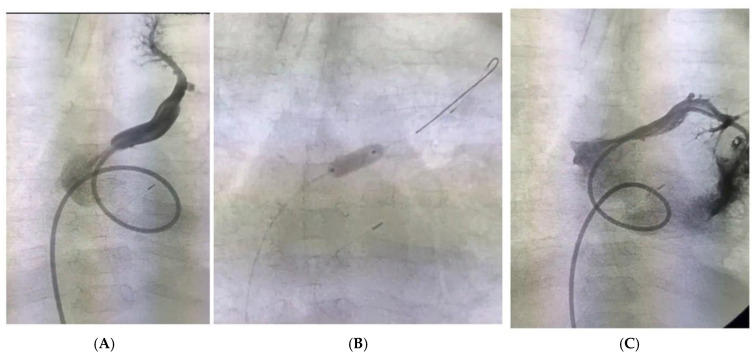
(**A**) Stenotic ostium of left upper pulmonary vein; (**B**) balloon venoplasty; (**C**) control angiography after dilatation.

After the follow-up period (32 months, IQR 8–99), an echocardiography examination highlighted the average diameters of the LV (EDD 34.5 mm, IQR 26.37–38.25) and LA (20.0 mm, IQR 16.0–26.0). Tricuspid insufficiency was mild (1+, IQR 1–1), with normal right chamber peak systolic pressure (22 mmHg, IQR 20–28). The velocity across pulmonary venous confluence was 0.8 m/s (IQR 0.77–1.62). Five patients had obstructive flow across the PVC in the LA (three were reoperated on). A smaller LV EDD was observed in those patients before the first operation (11.5 mm, IQR 11–12 mm vs. 14.25, IQR 12.1–16.7 mm; *p* = 0.04). Four were reoperated on—three due to postoperative obstruction between PVC and the LA (a sutureless technique in one), while in one patient, a redirection of the VCI was performed. In one patient, after the second procedure, percutaneous pulmonary balloon venoplasty was performed using a coronary balloon 5 × 15 mm (Figure 4). The mean pressure before the venoplasty was 19 mmHg, while afterward it was 9 mmHg. After the procedure, we started with sirolimus. Four patients had reentrant supraventricular tachycardia. Six patients had persistent VV; in four, VV was not ligated on operation, while in two, it was ligated below the left upper pulmonary vein inflow.

Four patients (three male and one female), aged 12.3 on average (IQR 8.9–14.7), underwent vertical vein embolization. One of those patients previously underwent an operation and reoperation. Supracardiac TAPVR had 3 patients, while one had a mixed type. None of the patients had associated CHD. The median body weight on occlusion was 48 kg (IQR 38.0–62.75). The pulmonary output (Qp:Qs) was 2.5 times (IQR 1.85–4.35) higher than systemic. The temporary balloon occlusion of the vertical vein was tolerated without a significant rise in the pulmonary mean pressure (the mean pressure before the test: 13.5 ± 2.1 mmHg; after the test: 14.7 ± 2.2 mmHg; *p* = 0.06). All patients achieved complete occlusion with AVP2; in one, AVP2 was 18 mm, and in three, AVP2 was 22 mm. In the female patient, AVP2 20 mm was implanted first, but the day after the implantation, the X-ray and echocardiography examination highlighted the device embolization in the right pulmonary branch. The device was extracted uneventfully percutaneously using the ONE Snare^®^ Endovascular Snare System. The complete occlusion was achieved with AVP2 22 mm on the subsequent heart catheterization. An X-ray confirmed the correct device position. During the follow-up period, the absence of flow across the VV was registered with the adequately placed device and a larger left heart than the right.

The LA diameter Z score was statistically significantly lower in the patients with persistent VV than those without (−4.9, IQR −5.75–3.9 vs. −2.7, IQR −4.3–−2; *p* = 0.032). The age during the operation and other echocardiographic parameters did not influence the persistence of VV in our patients (Table 2). The patients with an LA diameter Z score lower than −4 had frequent persistent VV (4/9; *p* = 0.001) and had an almost 19-times increased risk for VV persistence (OR 18.6, 95%CI 1.6–216.0). Two patients with VV persistence due to ligation below the left upper pulmonary vein inflow were not included in this analysis, but their LA diameter Z scores were −2 and −3.8, respectively.

## 4. Discussion

Preoperative pulmonary venous obstruction (PVO), mixed anatomic variations, single ventricular physiology, and heterotaxy remain important risk factors for poor postoperative survival [10]. The mortality rate was 17.5% in our cohort, and five out of seven patients had combined CHD. Obstruction after the surgical correction was observed in 12.5%; three out of five patients underwent the reoperation, while balloon valvuloplasty was performed on one. According to the literature data, reoperation is required in approximately 10 to 15% of patients with isolated TAPVR due to PVO ± confluence stenosis [10]. Stenosis usually develops within 6 months of primary repair [11]. Pathological findings of PVO after surgery are thickened intimal tissues and a proliferation of fibrous or elastic tissues. It is likely that the intimal hyperplasia process starts at the anastomotic suture site and extends towards the pulmonary ostia located very close by [11]. Although balloon angioplasty achieved stenosis release, the improvement is only temporary, with a high likelihood of restenosis, and many cases involve the procedure being performed multiple times [12]. The studies concluded that adding sirolimus in patients with moderate-severe PVO helps decrease disease progression with a reduced frequency of interventions [13]. Consequently, our patient started rapamycin therapy after the balloon venoplasty of the left upper pulmonary vein.

Hoashi et al. found that patients with TAPVR who had significantly obstructed or completely occluded venous pathways drainage needed prolonged nitric oxide inhalation and mechanical ventilation after the operation, required frequent reoperations during the in-hospital stay, developed frequent postoperative PVS, and showed poor overall survival rates [14]. Additionally, Frommelt et al. concluded that patients with the obstruction had a poorer outcome with a significantly increased need for ECMO support, late PVO, and mortality rate [15]. In our study, the obstruction did not influence the mortality rate and reoperations.

Repairing supracardiac TAPVR involves a side-to-side anastomosis between the common pulmonary venous sinus and the LA, with ASD closure, and many surgeons prefer to ligate the VV to prevent residual left-to-right shunts. In 1997, Cope et al. suggested that leaving the unligated VV was a safe option and that it may be expected to close spontaneously in most cases [6]. In 2001, Kumar et al. reported a contradictory finding, suggesting that an unligated VV will likely require subsequent surgical/intervention closure [16]. Kobayashi et al. presented a patient with mixed TAPVR in whom temporary occlusion of the VV during surgery resulted in severe hypoxemia along with severe mitral regurgitation. After the release of the VV, oxygenation improved with only a mild mitral regurgitation. Consequently, they concluded that the deterioration was caused by a poorly compliant left-sided chamber and left the VV open [17]. However, in patients with small left chambers and inadequate compliance of LA, not ligating the VV as an import-decompression conduit may improve survival as it provides transient decompression of the right side of the heart for pulmonary hypertension crises in the immediate postoperative period [10,18]. We found that an LA diameter Z score lower than −4 increased the risk for VV persistence by almost 19 times.

The VV remains patent more often in supracardiac TAPVR [8]. Interestingly, half of the patients had persistent unligated vertical veins [17]. In our case series, three out of four patients had a supracardiac type, while one had a mixed type.

In most cases, the VV spontaneously closes after surgery due to the preferential flow to the LA and increased compliance [7,18]. From a literature review, the prevalence of unligated VV, which later produces clinically relevant left-right shunts, seems rare. A patent unligated or partially VV presents as a pretricuspid shunt, producing right heart dilatation [18]. The first reports of percutaneous VV closure were published in 1992, with Hausdorf using an 11F Rashkind PDA Occluder system. After this case, few reports of transcatheter occlusion of the unligated vertical vein after TAPVR surgery were published [7,8,10,11,17,19,20,21]. We, as well as all authors, performed the balloon test occlusion before the VV occlusion due to the potential restrictive physiology of the left heart chambers [7,8,10,11,17,19,20,21]. All our patients achieved VV embolization with AVP2. Other authors use the Rashkind PDA Occluder, Amplatzer duct occluder, AVP1, AVP2, Amplatzer Muscular VSD occluder, Gianturco coil and “Cera” vascular plugs [7,8,10,11,17,19,20,21].

We had only one complication during the VV occlusion. In one patient, the AVP2 20 mm was embolized after the VV occlusion into the right pulmonary artery. The device was percutaneously extracted with a snare catheter.

Due to the possibility of VV persistence after the TAPVR operation, it is reasonable to ligate it during the operation. After the connection of the LA and pulmonary venous confluence, but before the VV ligation, the previous test temporary occlusion should be performed with manometric measurement in LA and PVC. This careful testing ensures that the surgical approach is tailored to the patient’s needs and conditions. If the pressure increases in PVC, it is most likely that surgical anastomosis is stenotic. This should be confirmed by transesophageal echocardiography. On the other hand, if the LA pressure increases during the test, VV should not be ligated. Special care is needed in case the VV is not ligated in patients with an LA Z score lower than −4 because they are at an almost 19-times higher risk of VV persistence. In this patient group, a careful echocardiography examination with a particular reference to the persistence of VV should be routinely performed.

While our study provides valuable insights, it is important to acknowledge its limitations. The retrospective design and the reliance on medical records for data collection are areas that warrant further investigation. Additionally, the relatively small number of patients in our cohort underscores the need for larger-scale studies to validate our findings and draw more robust conclusions. It is necessary to conduct randomized multicenter studies to establish risk factors for VV persistence with a significant level of recommendations.

## 5. Conclusions

Six patients had persistent VV; in four, VV was not ligated on operation, while in two, it was ligated below the left upper pulmonary vein inflow. We found that an LA diameter Z score lower than −4 increased the risk for VV persistence by almost 19 times. Four underwent vertical vein embolization with the previous balloon test occlusion. All patients achieved complete VV occlusion with AVP2, while one had a complication.

In our cohort, one-fifth had associated anomalies. In this patient’s group, the mortality rate was higher. Six patients had obstructive flow across the pulmonary venous confluence in the left atrium. Four were reoperated on (a sutureless technique in one), and one underwent percutaneous pulmonary balloon venoplasty.

## Figures and Tables

**Table 1:** Frequency of total anomalous pulmonary vein return types and associated congenital heart defects.

	Number of Patients (*n*)	Percent (%)
TAPVR male	26	65
TAPVR type	40	100
Supracardiac	19	47
Cardiac	7	17.5
Infracardiac	4	22.5
Mixed	5	12.5
TAPVR obstruction	10	25
TAPVR associated CHD	8	20
AV defect, L-TGA, AAP	1	2.5
CoA, inlet VSD, Ao stenosis	1	2.5
CoA, VCSSP	1	2.5
AV defect, AAP; VCSSP, dextrocardia	1	2.5
Univentricular heart, AV defect, L-TGA, VCSSP	1	2.5
Univentricular heart, D-TGA, VSD	1	2.5
AV defect, D-TGA, AAP, VCSSP	1	2.5
Cor triatriatum, AP stenosis, VCSSP	1	2.5

Abbreviations: TAPVR—total anomalous pulmonary venous return, CHD—congenital heart defects, AV—atrioventricular canal, L-TGA—L transposition of the great vessels, AAP—pulmonary artery atresia, CoA—aortic coarctation, VCSSP—persistent left superior vena cava, D-TGA—D transposition of the great vessels, VSD—ventricular septal defect, AP—pulmonary artery, Ao—aorta.

**Table 2:** Demographic and echocardiographic data for the patients and the difference between the patients with and without persistent vertical vein.

	All No = 40	VV No = 4	No VV No = 36	*p* Value
TAPVR diagnosis (DOL)	2 (IQR 1–7.7)	12 (IQR 1.5–72.75)	2 (IQR 1–6.75)	0.44
TAPVR operation (DOL)	8 (IQR 5–57)	18.5 (IQR 7.75–77.2)	7 (IQR 4–57)	0.39
TAPVR operation (BW)	3.3 (IQR 2.85–4.0)	3.6 (IQR 3.1–5.5)	3.3 (IQR 2.8–4.0)	0.44
Preoperative ECHO				
LV EDD (mm)	13.0 (IQR 12–16)	14.5 (IQR 12.2–19.0)	13.0 (IQR 12–16)	0.38
LV EDD Z score	−3.85 (IQR −4.97–−1.32)	−2.55 (IQR −4.2–2.25)	−3.8 (IQR 5.0–−1.9)	0.25
LA diameter (mm)	8 (IQR 6.5–9)	6 (IQR 6–6.7)	8 (IQR 7–9)	0.018
LA diameter Z score	−3.1 (IQR −4.7–−2.2)	−4.9 (IQR −5.7–−3.9)	−2.7 (IQR −4.3–−2.0)	0.013
Mitral insufficiency (+)	0 (IQR 0–0)	0 (IQR 0–0)	0 (IQR 0–0)	0.68
Tricuspid insufficiency (+)	1.0 (IQR 0.5–1)	0.5 (IQR 0–1.5)	1 (IQR 0.5–1)	0.29
Tricuspid pressure gradient (mmHg)	38 (IQR 32–56)	32 (IQR 29–32)	44.5 (IQR 33.5–59.0)	0.36
ASD diameter (mm)	4 (IQR 2–6.5)	3.5 (IQR 2.7–5)	5.0 (IQR 2–7)	0.42

Abbreviations: VV—vertical vein, DOL—day of life, TAPVR—total anomalous pulmonary venous return, BW—body weight, LV—left ventricle, EDD—end-diastolic diameter, LA—left atrium, ASD—atrial septal defect.

## Data Availability

Data are contained within the article.

## References

[1] Luca A.-C., Curpăn A., Manea R.-S., Butnariu L.I., Țarcă E., Starcea I.M., Roșu S.T., Mîndru D.E., Macsim E., Adumitrăchioaiei H. (2023). Total Anomalous Pulmonary Venous Return in the Time of SARS-CoV-2-Case Report. Children.

[2] Kao C.C., Hsieh C.C., Cheng P.J., Chiang C.H., Huang S.Y. (2017). Total Anomalous Pulmonary Venous Connection: From Embryology to a Prenatal Ultrasound Diagnostic Update. J. Med. Ultrasound.

[3] Cooley D.A., Cabello O.V., Preciado F.M. (2008). Repair of total anomalous pulmonary venous return: Results after 47 years. Tex. Heart Inst. J..

[4] Li G., Meng B., Zhang C., Zhang W., Zhou X., Zhang Q., Ding Y. (2023). Total anomalous pulmonary venous connection in 80 patients: Primary sutureless repair and outcomes. Front. Surg..

[5] Thanh D.Q.L., Giau H.T.N., Huong T.N.G., Linh T.N.U., Phuc V.M., Vuong N.L. (2022). Sutureless Closure Versus Conventional Technique in the Primary Surgery of Total Anomalous Pulmonary Venous Connection: A Systematic Review and Meta-analysis. Pediatr. Cardiol..

[6] Cope J.T., Banks D., McDaniel N.L., Shockey K.S., Nolan S.P., Kron I.L. (1997). Is vertical vein ligation necessary in repair of total anomalous pulmonary venous connection?. Ann. Thorac. Surg..

[7] Charbel R., Hanna N., Daou L., Saliba Z. (2018). Catheter closure of a recanalized vertical vein after repair of total anomalous pulmonary venous connection. Clin. Case Rep..

[8] Verma S., Subramanian A., Saileela R., Koneti N.R. (2015). Transcatheter closure of patent vertical vein after repair of total anomalous pulmonary venous connection. Ann. Pediatr. Cardiol..

[9] Lopez L., Frommelt P.C., Colan S.D., Trachtenberg F.L., Gongwer R., Stylianou M., Bhat A., Burns K.M., Cohen M.S., Dragulescu A. (2021). Pediatric Heart Network Investigators. Pediatric Heart Network Echocardiographic Z Scores: Comparison with Other Published Models. J. Am. Soc. Echocardiogr..

[10] Catalán Cabrera A., Condori Alvino K. (2023). Cierre percutáneo de vena vertical, posterior a la reparación de conexión anómala pulmonar total supracardiaca, usando oclusor para defecto del septum atrial. Reporte de caso. Arch. Peru. Cardiol. Cir. Cardiovasc..

[11] Jain S., Bachani N.S., Pinto R.J., Dalvi B.V. (2018). Dual pathology causing severe pulmonary hypertension following surgical repair of total anomalous pulmonary venous connection: Successful outcome following serial transcatheter interventions. Ann. Pediatr. Cardiol..

[12] Kurita Y., Baba K., Kondo M., Eitoku T., Kasahara S., Iwasaki T., Ohtsuki S., Tsukahara H. (2019). Clinical outcomes after the endovascular treatments of pulmonary vein stenosis in patients with congenital heart disease. Cardiol. Young.

[13] Shorofsky M.J., Morgan G.J., Mejia E., Rodriguez S.A., Greene M., Sheaks P., Ivy D., Zablah J.E. (2023). Management of Complex Pulmonary Vein Stenosis at Altitude Combining Comprehensive Percutaneous Interventional Treatment with Sirolimus, Pulmonary Hypertension Medications and Intraluminal Imaging with Optical Coherence Tomography. Pediatr. Cardiol..

[14] Hoashi T., Kagisaki K., Kurosaki K., Kitano M., Shiraishi I., Ichikawa H. (2014). Intrinsic obstruction in pulmonary venous drainage pathway is associated with poor surgical outcomes in patients with total anomalous pulmonary venous connection. Pediatr. Cardiol..

[15] Frommelt P.C., Sheridan D.C., Deatsman S., Yan K., Simpson P., Frommelt M.A., Litwin S.B., Tweddell J.S. (2010). Unobstructive total anomalous pulmonary venous return: Impact of early elective repair on the need for prolonged mechanical ventilatory support. Pediatr. Cardiol..

[16] Kumar RN S., Dharmapuram A.K., Rao I.M., Gopalakrishnan V.C., Pillai V.R., Nazer Y.A., Cartmill T. (2001). The fate of the unligated vertical vein after surgical correction of TAPVC in early infancy. J. Thorac. Cardiovasc. Surg..

[17] Kobayashi D., Forbes T.J., Delius R.E., Aggarwal S. (2012). Amplatzer vascular plug for transcatheter closure of persistent unligated vertical vein after repair of infracardiac total anomalous pulmonary venous connection. Catheter. Cardiovasc. Interv..

[18] Mulia E.P.B., Rahman M.A. (2023). Treatment considerations in total anomalous pulmonary venous connection. Proc. Singap. Healthc..

[19] Lombardi M., Tagliente M.R., Pirolo T., Massari E., Sisto M., Vairo U. (2016). Transcatheter closure of an unligated vertical vein with an Amplatzer Vascular Plug-II device. J. Cardiovasc. Med..

[20] Narula N., Wilson N., Kumar R.S. (2007). Transcatheter closure of persistent unligated vertical vein after TAPVC surgery using the Amplatzer PDA device. Catheter. Cardiovasc. Interv..

[21] Pascual-Tejerina V., Sánchez-Recalde Á., Gutiérrez-Larraya F., Ruiz-Cantador J., Rodríguez-Padial L., Zamorano J.L. (2021). Transcatheter closure of a vertical vein in a patient with total anomalous pulmonary venous drainage decompressing the left atrium with an AFR device. Rev. Española De Cardiol..

